# Supplementary material for: Reconstitution of multistep recruitment of ULK1 to membranes in autophagy
Source: Sci Adv. 2026 Sep 4;12(36):eaeg3201. doi: 10.1126/sciadv.aeg3201 (PMC13544240; doi:10.1126/sciadv.aeg3201)
Supplement: Supplementary file 1 [file sciadv.aeg3201_sm.pdf]

Science Advances
AAAS

Supplementary Materials for

Reconstitution of multistep recruitment of ULK1 to membranes in autophagy

Yongjia Duan *et al.*

Corresponding author: Ian G. Ganley, i.ganley@dundee.ac.uk;
Gerhard Hummer, gerhard.hummer@biophys.mpg.de; James H. Hurley, jimhurley@berkeley.edu

Sci. Adv. **12**, eaeg3201 (2026)
DOI: 10.1126/sciadv.aeg3201

This PDF file includes:

Figs. S1 to S10
Table S1

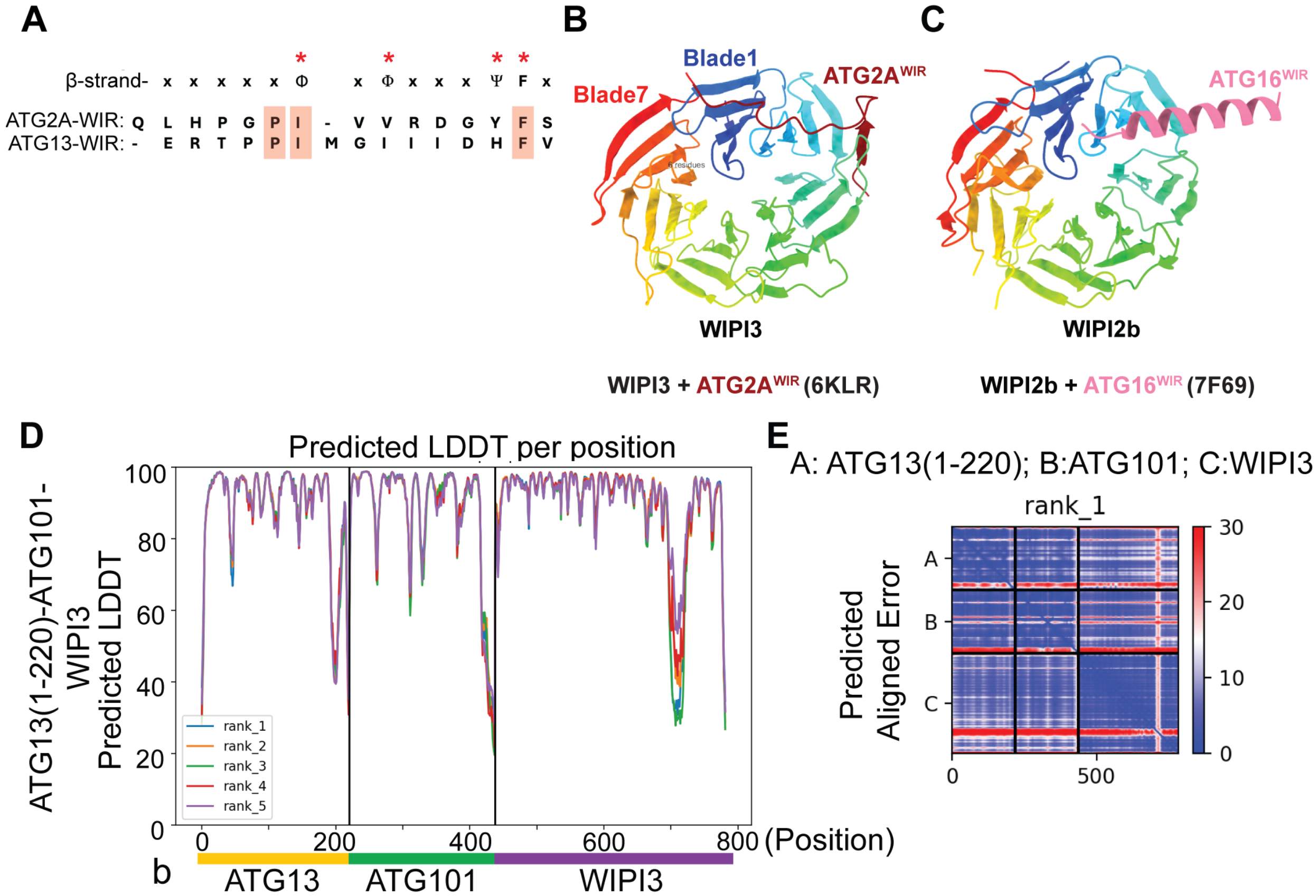

Figure S1. Comparison of the predicted binding between WIPI effectors and Structure prediction confidence of ATG13-ATG101-WIPI3

(A) Comparison of human ATG2-WIR and ATG13-WIR. Φ is a hydrophobic residue and Ψ is an aromatic residue. **(B)** Crystal structure (PDB: 6KLR) of the ATG2A WIR motif bound to WIPI3 in the pockets between blades1 and 2, and blades2 and 3. **(C)** Crystal structure (PDB: 7F69) of ATG16 W2IR motif bound to WIPI2 at the cognate blade2 and 3 pocket of WIPI2. (**D, E**) pLDDT (D) and PAE (E) of Alphafold model of ATG13-ATG101-WIPI3 in Fig. 1C. Colab AlphaFold provided 5 models (rank_1-rank_5), rank_1 model was chosen to show in Fig. 1C.

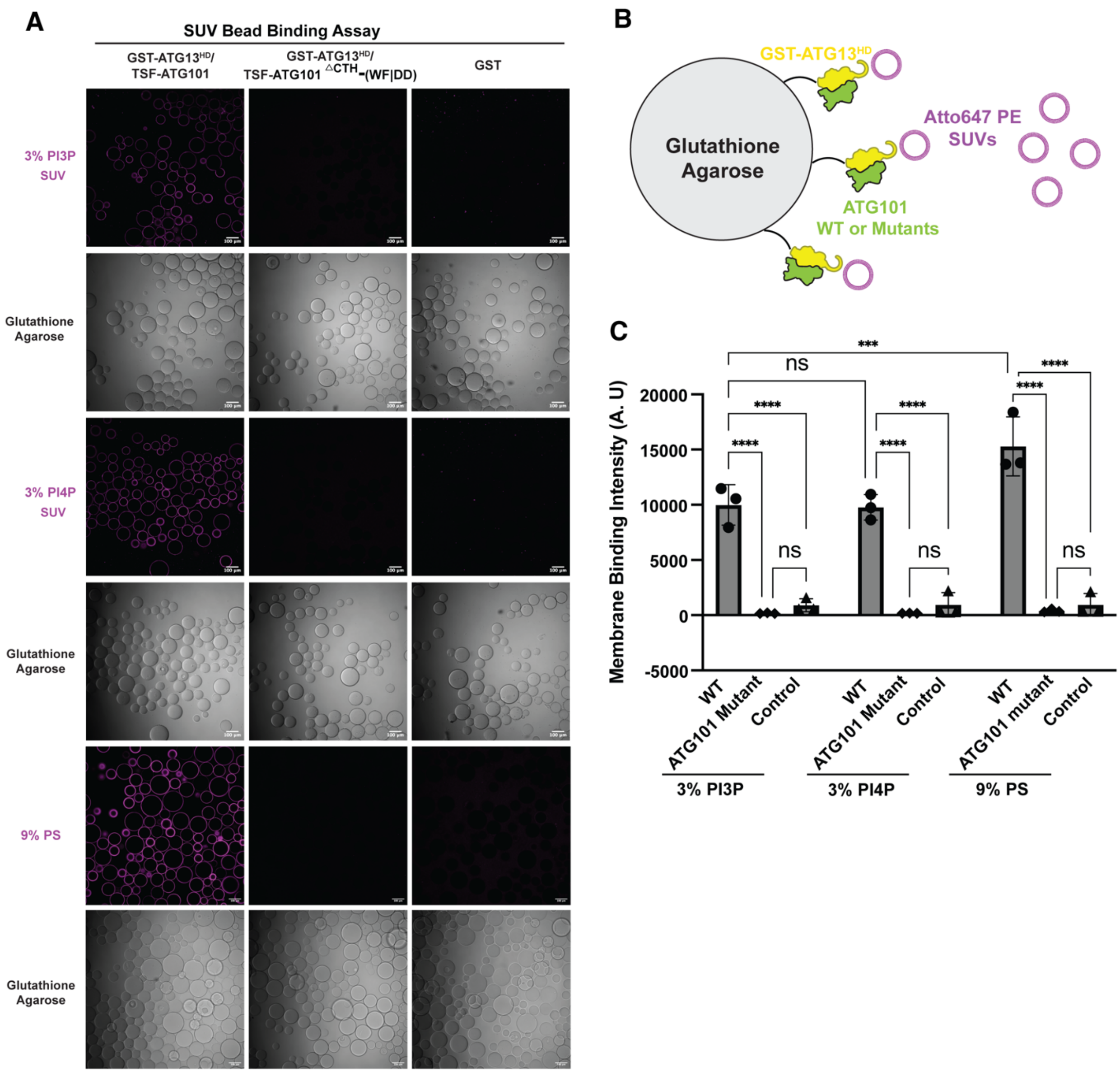

Figure S2: ATG13:ATG101 HORMA recruited to high curvature membrane by SUV-bead binding assay

(A) Fluorescent images show the results of GST beads binding experiments between GST-ATG13/ATG101 HORMA and SUVs with 3% PI3P, 3% PI4P or 9% PS. The scale bar is 100 µm. **(B)** Schematic of SUV and GST bead binding assay. ATG13:ATG101 HD was included at 1 µM concentration, and lipids were included at 100 µM. **(C)** Quantification of SUV binding to glutathione agarose beads as recruited by GST tagged ATG13:ATG101 WT or ATG13:ATG101 mutant. Three biological replicates were performed, with at least three images taken for each

replicate. The average fluorescence intensity of all beads in each image was calculated. ***$P<0.001$, ****$P<0.0001$, ns, non-significance.

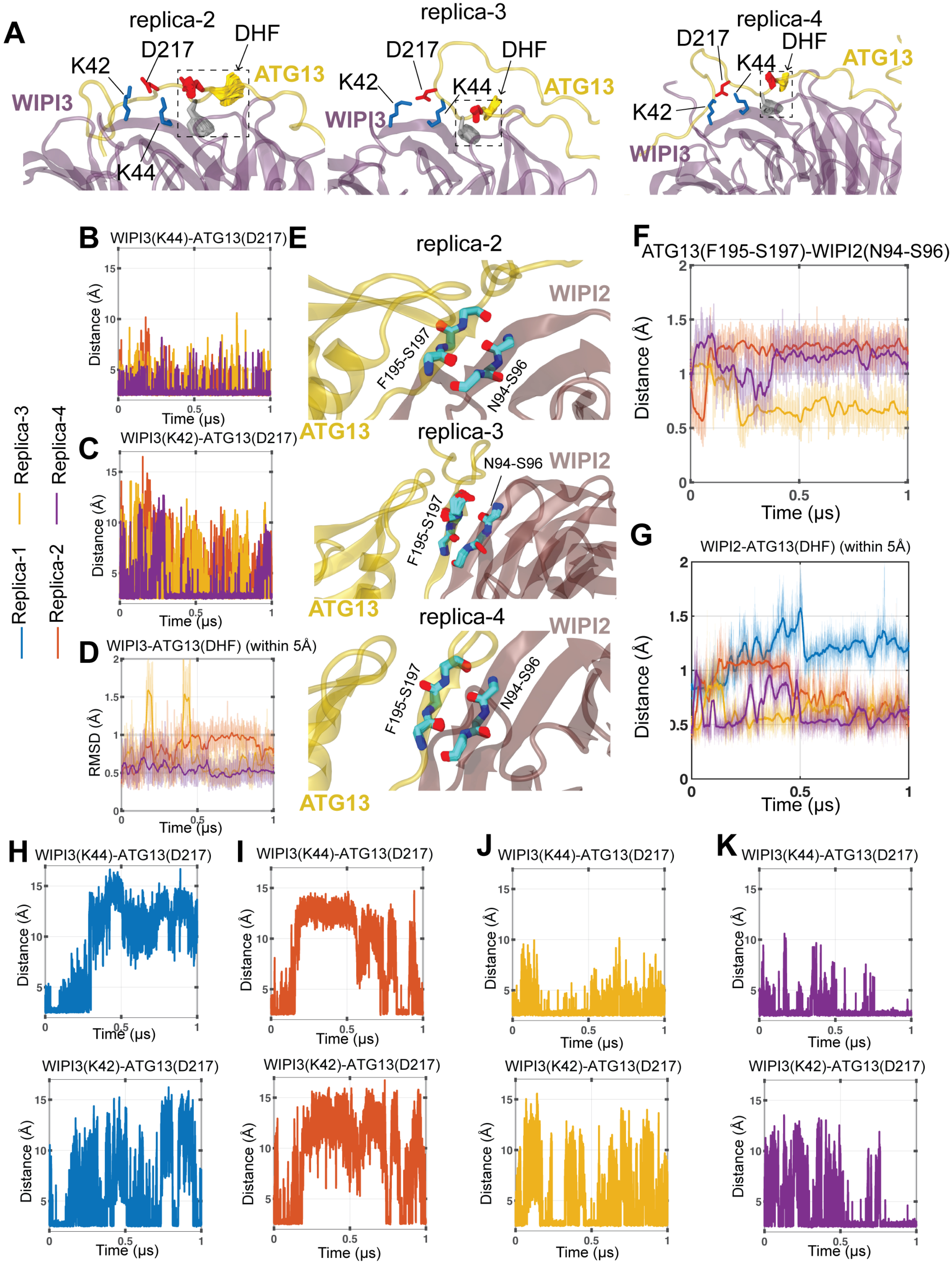
A
replica-2
replica-3
replica-4
K42
D217
DHF
K44
WIPI3
ATG13
B
WIPI3(K44)-ATG13(D217)
C
WIPI3(K42)-ATG13(D217)
D
WIPI3-ATG13(DHF) (within 5Å)
RMSD (Å)
Distance (Å)
Time (μs)
Replica-1
Replica-2
Replica-3
Replica-4
E
WIPI2
F195-S197
N94-S96
F
ATG13(F195-S197)-WIPI2(N94-S96)
G
WIPI2-ATG13(DHF) (within 5Å)
H
I
J
K

Figure S3. Dynamics of membrane-bound ATG13–ATG101–WIPI3 and ATG13–ATG101–WIPI3–WIPI2 complexes across multiple replica simulations.

(A) Zoomed- in view of the ATG13–WIPI3 interface (as shown in Fig. 3B for replica 1) in replicas 2, 3, and 4 of the ATG13–ATG101–WIPI3 simulation. **(B, C)** Time evolution of the minimum distance between the OD1/OD2 atoms of ATG13 D217 and the NZ atom of WIPI3 K44 (B) or K42 (C) in replicas 2–4 (replica 1 shown in Fig. 3C, 3D). **(D)** Backbone RMSD of the ATG13 DHF motif and WIPI3 residues within 5 Å of this region for replicas 2–4 (replica 1 shown in Fig. 3E). **(E)** Zoomed-in views of the ATG13–WIPI2 interface in replicas 2, 3, and 4 of the ATG13–ATG101–WIPI3–WIPI2 simulation. **(F)** Backbone RMSD of the residues shown in (F) as a function of simulation time across different replicas (replica 1 shown in Fig. 3I). **(G)** Backbone RMSD evolution of the ATG13–WIPI3 interface (as defined in D for ATG13-ATG101- WIPI3) in different replicas of the ATG13–ATG101–WIPI3–WIPI2 simulation. **(H-K)** Time evolution of the minimum distance between the OD1/OD2 atoms of ATG13 D217 and the NZ atoms of WIPI3 K44 and K42 in replicas 1 (H), 2 (I), 3 (J), and 4 (K) of the ATG13–ATG101–WIPI3–WIPI2 simulation.

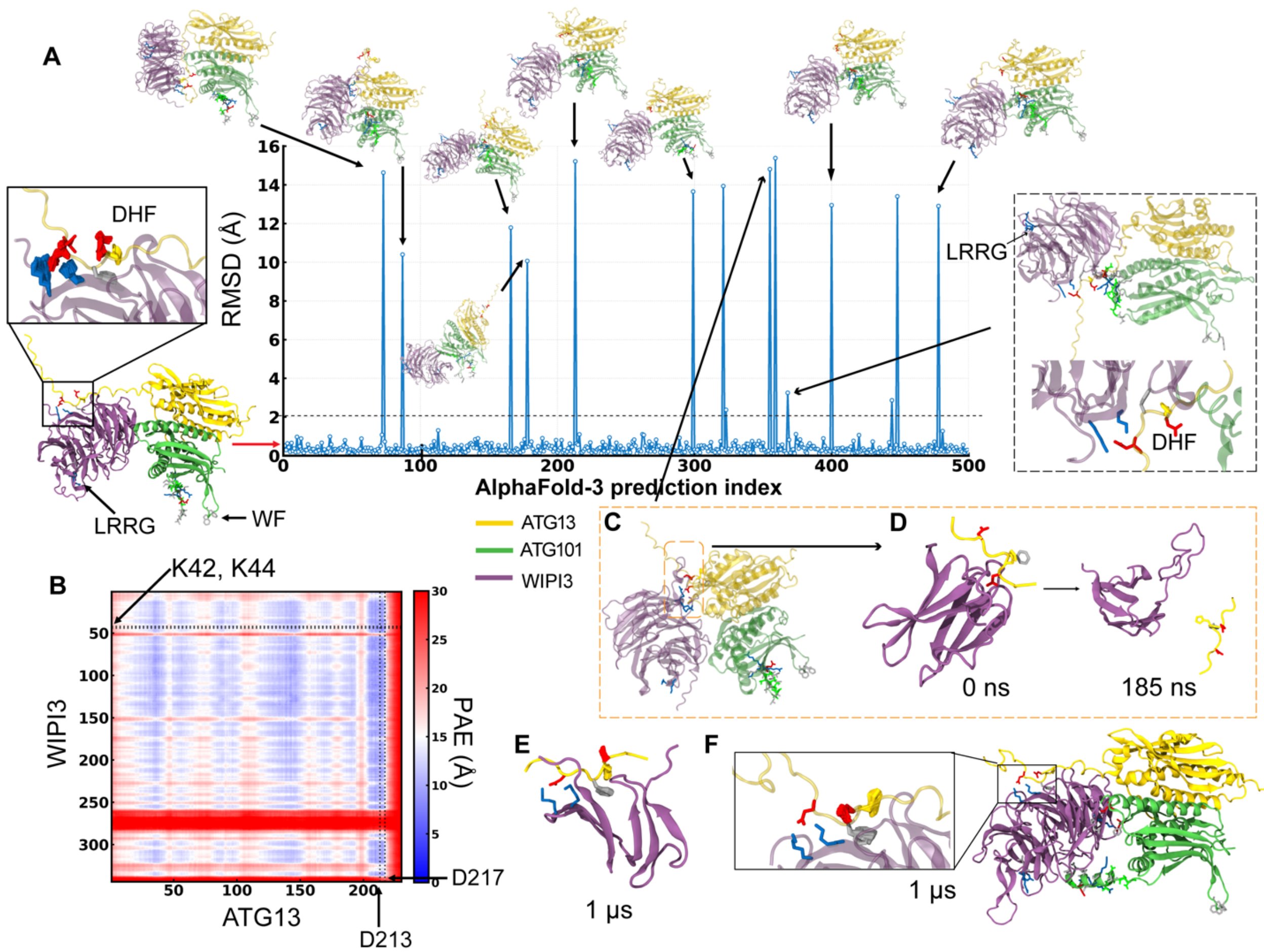

Figure S4: Structural validation of the ATG13-ATG101-WIPI3 interface using multiple AlphaFold-3 iterations and stability analysis of ATG13-WIPI3 interface fragments using MD simulations.

(A) RMSD analysis of the ATG13-WIPI3 interface relative to the reference structure in Fig. 3. Key interface residues—including the ATG13 DHF motif and D217, the WIPI3 K42–K44 residues and LRRG motif, and the ATG101 WF finger—are rendered as sticks. The inset displays the superimposed coordinates of the DHF motif for all models within this primary cluster. Outlier structures (RMSD > 2 Å) are presented. The model within the black box exhibits a conserved ATG13-WIPI3 interface (zoomed in view below) but features a flipped LRRG motif relative to the WF finger. **(B)** PAE scores for the WIPI3-ATG13 interface, demonstrating high-confidence predictions centered on the DHF motif. **(C, D)** A specific outlier conformation model in A (C) and its interface fragment (D). Dissociation of this fragment occurred within 185 ns. **(E)** The ATG13-WIPI3 interface from the dominant cluster remains stable throughout the 1 μs simulation;

sidechains of the DHF motif are shown superimposed across all trajectory frames. **(F)** Stability of the full hetero-trimeric complex from the dominant cluster, highlighting the preserved D217–K42/K44 salt bridge and stable binding of the DHF motif during the 1 μs membrane-free simulation.

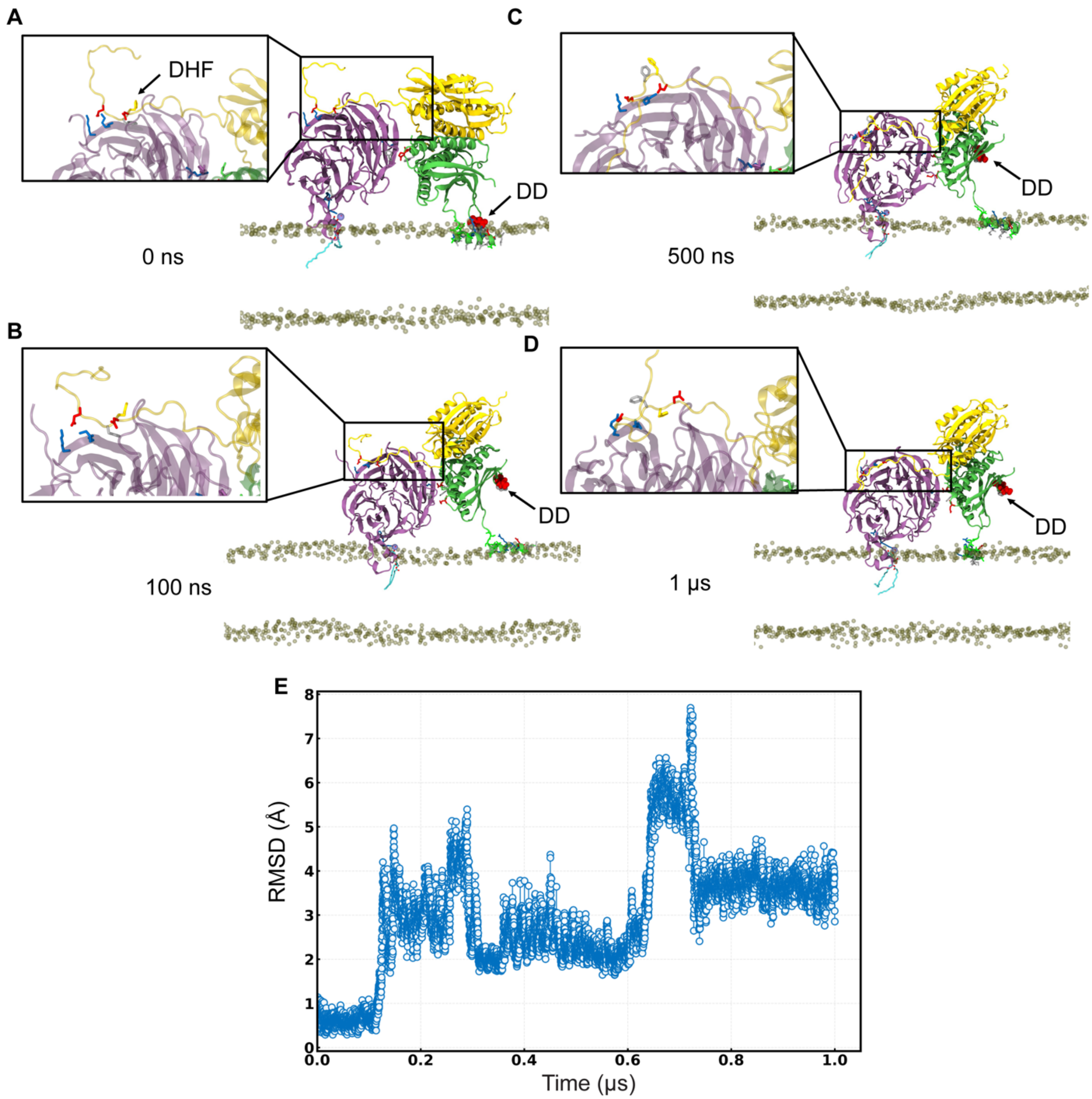

Figure S5: Impact of the ATG101 WF-to-DD mutation on the stability and membrane association of the ATG13-ATG101-WIPI3 complex.

(A-D) Snapshot of the complex in the presence of the membrane (only phosphate groups are shown for clarity) at (A) 0 ns (B) 100 ns (C) 500 ns and (D) 1 μs. The newly introduced DD finger of ATG101 reverts to a solvent-exposed state away from the membrane. Insets show a magnified view of the ATG13-WIPI3 interface, with key residues (DHF motif and D217 of ATG13; K42/K44 of WIPI3) shown as sticks. **(E)** RMSD evolution of the DHF motif of ATG13 and its nearby WIPI3 residues exhibit a significant destabilization of the interface due to the WF to DD mutation.

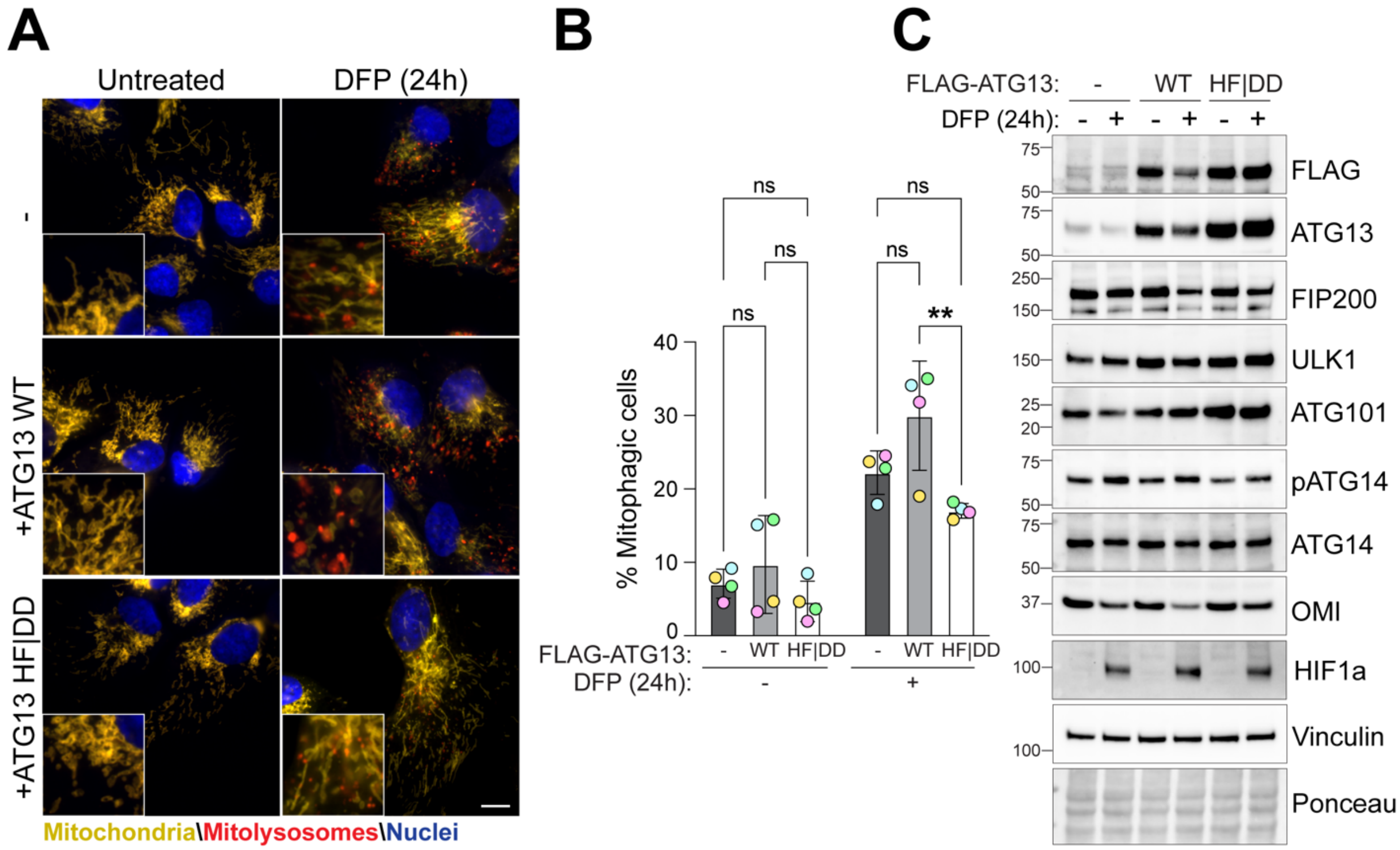

Figure S6: Effect of ATG13 HF|DD mutation on mitophagy

(**A**) Representative wide-field images of ARPE-19 cells stably expressing WT or HF|DD mutant FLAG tagged ATG13, and the *mito*-QC reporter. Cells were treated with 1mM DFP for 24 hours prior to imaging. (**B**) Flow cytometry analysis of the mCherry/GFP ratio (n = 4 biological replicates); statistics: two-way ANOVA with Tukey's multiple-comparisons test. **(C)** Representative immunoblots of the indicated proteins from cells as in (A). **P<0.01, ns, non-significance.

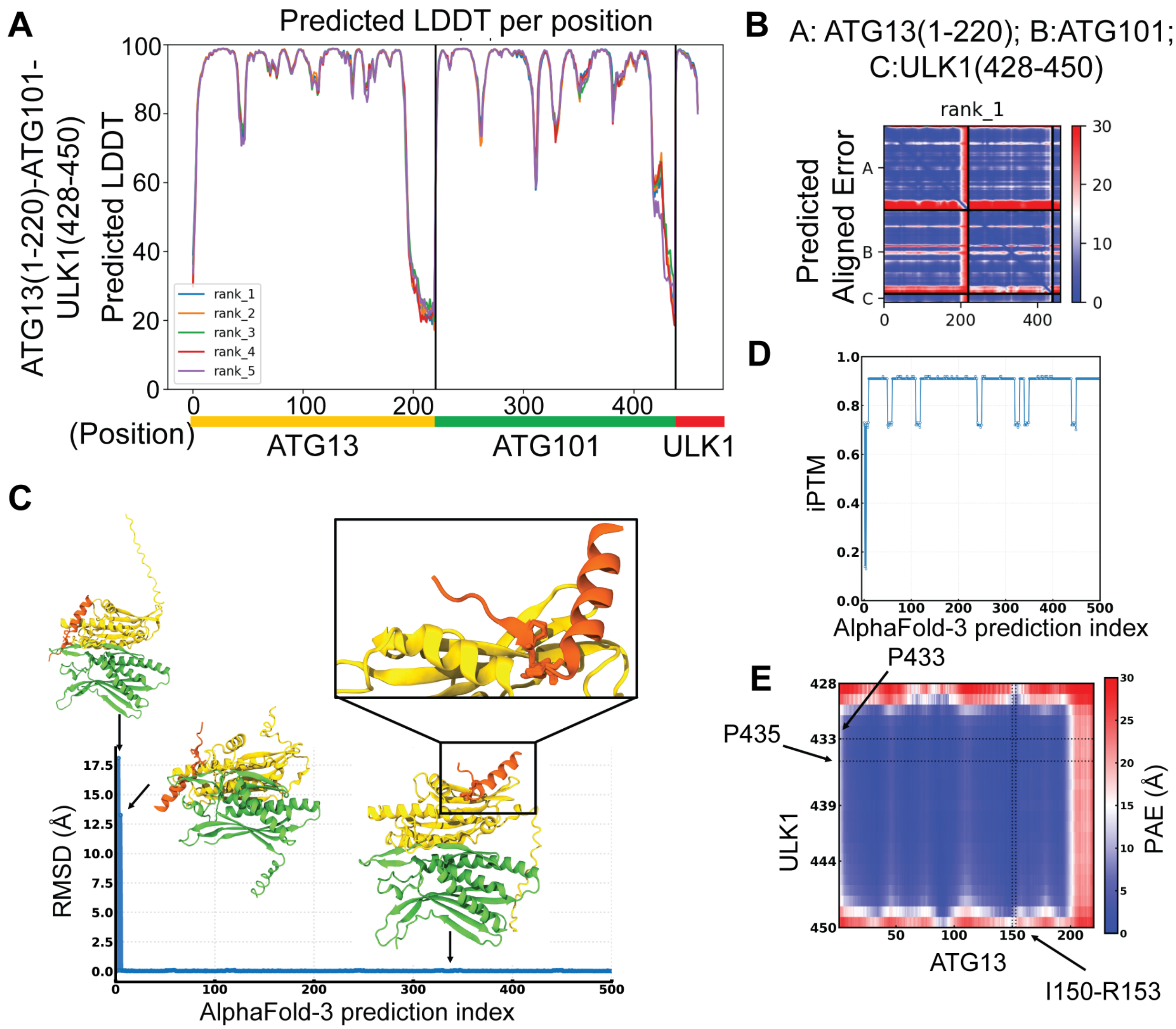

Figure S7: Structure prediction confidence of ATG13-ATG101-ULK1

(A, B) pLDDT (A) and PAE (B) of Alphafold model of ATG13-ATG101-ULK1 in Fig. 5C. Colab AlphaFold provided 5 models (rank_1-rank_5), rank_1 model was chosen to show in Fig. 5C. **(C)** RMSD analysis of the ULK1 (residues 428–450)–ATG13 (residues 150–153) interface relative to the reference structure in Fig. 5C identifies only two outliers (representative snapshot shown) among 500 total predictions. A snapshot of the dominant prediction is shown, with key ULK1 residues (P433, V434, P435) displayed as sticks. The inset shows the superposition of the PVP motif coordinates across all models within the primary cluster. **(D)** iPTM scores for all predicted ULK1 IDR–ATG13 models show consistently high values, with the exception of the outliers. **(E)** PAE score of a representative ULK1 IDR–ATG13 interface indicates high confidence, reflected

by low PAE values. ULK1 residues P433 and P435 are highlighted with dotted lines, showing contacts with ATG13 residues I150–R153 (indicated by a dotted vertical line).

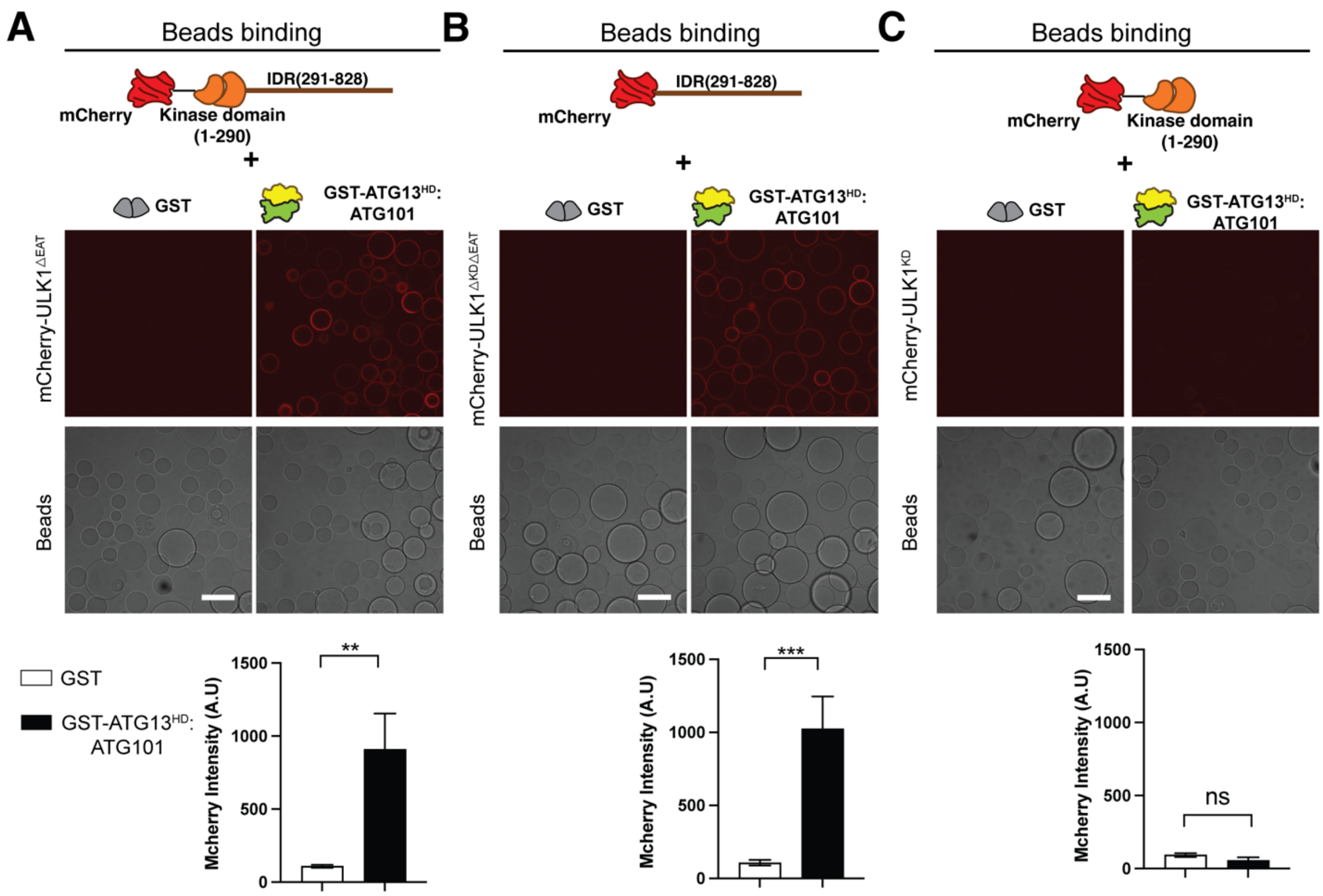

Figure S8. The IDR of ULK1 binds to ATG13(HORMA)/ATG101 directly

(A) ULK1 constructs lacking the EAT domain can bind to GST-ATG13^{HD}:ATG101, the bar graph in below showed the quantification of intensity of mcherry channel per bead; **(B)** ULK1-IDR maintain the ability that bind to GST-ATG13^{HD}:ATG101, the quantification showed in below. **(C)** ULK1^{KD} lost its binding to GST-ATG13^{HD}:ATG101. n=3 biological replicates, statistics: Unpaired t-test. Scale bar is 100 µm. Mean ± SEM was shown. **P<0.01, ***P<0.001, ns, non-significance.

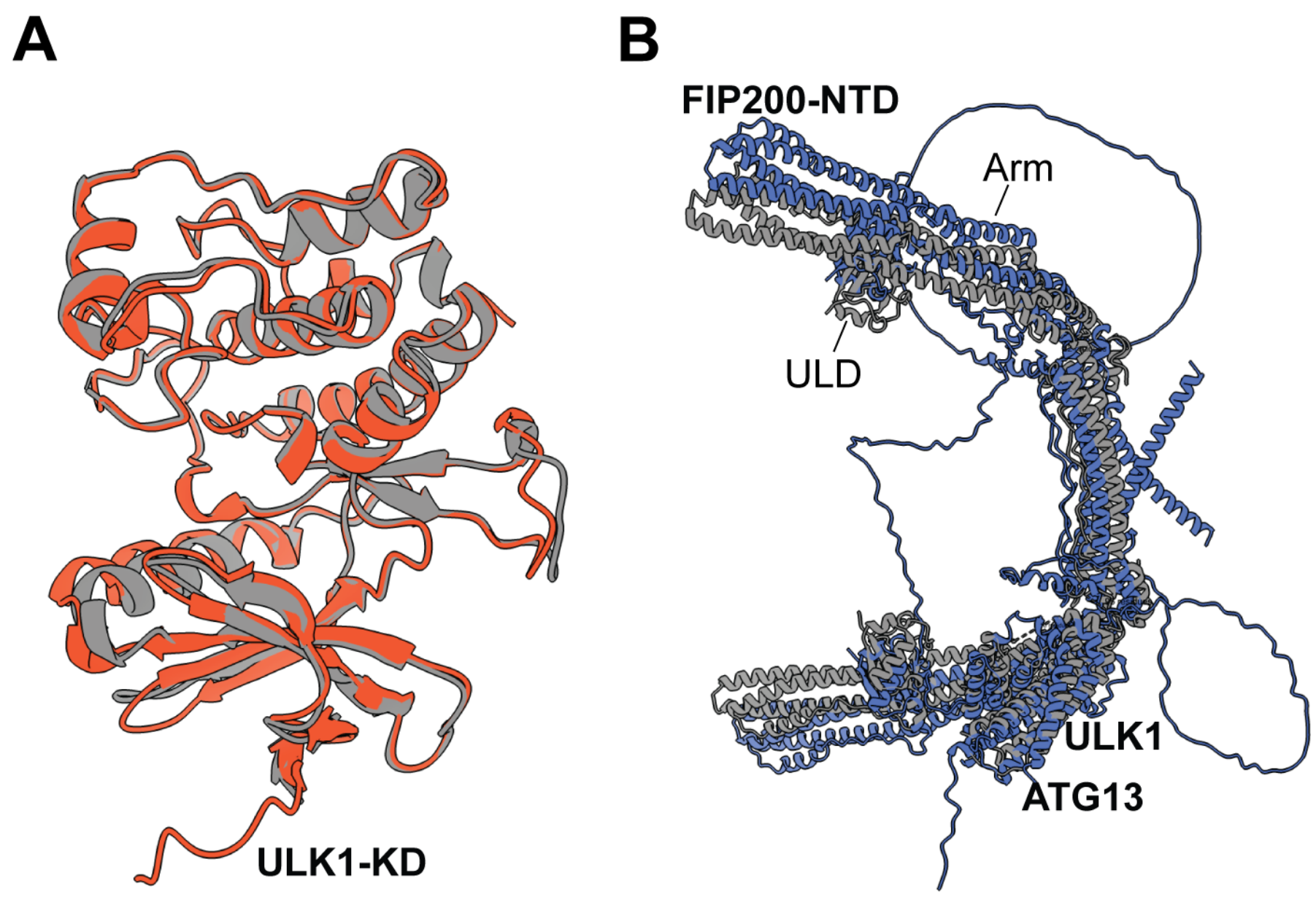

Figure S9: AlphaFold2 models used of ULK1-KD and ULK1C core.

(A) Overlay of ULK1-KD AlphaFold2 prediction (orange) with PDB ID 4WNO (grey). C_a RMSD for experimentally resolved residues is 1.3 Å. **(B)** Overlay of ULK1C core AlphaFold2 prediction (blue) with PDB ID 8SOI (grey). C_a RMSD for experimentally resolved residues is 9.6 Å.

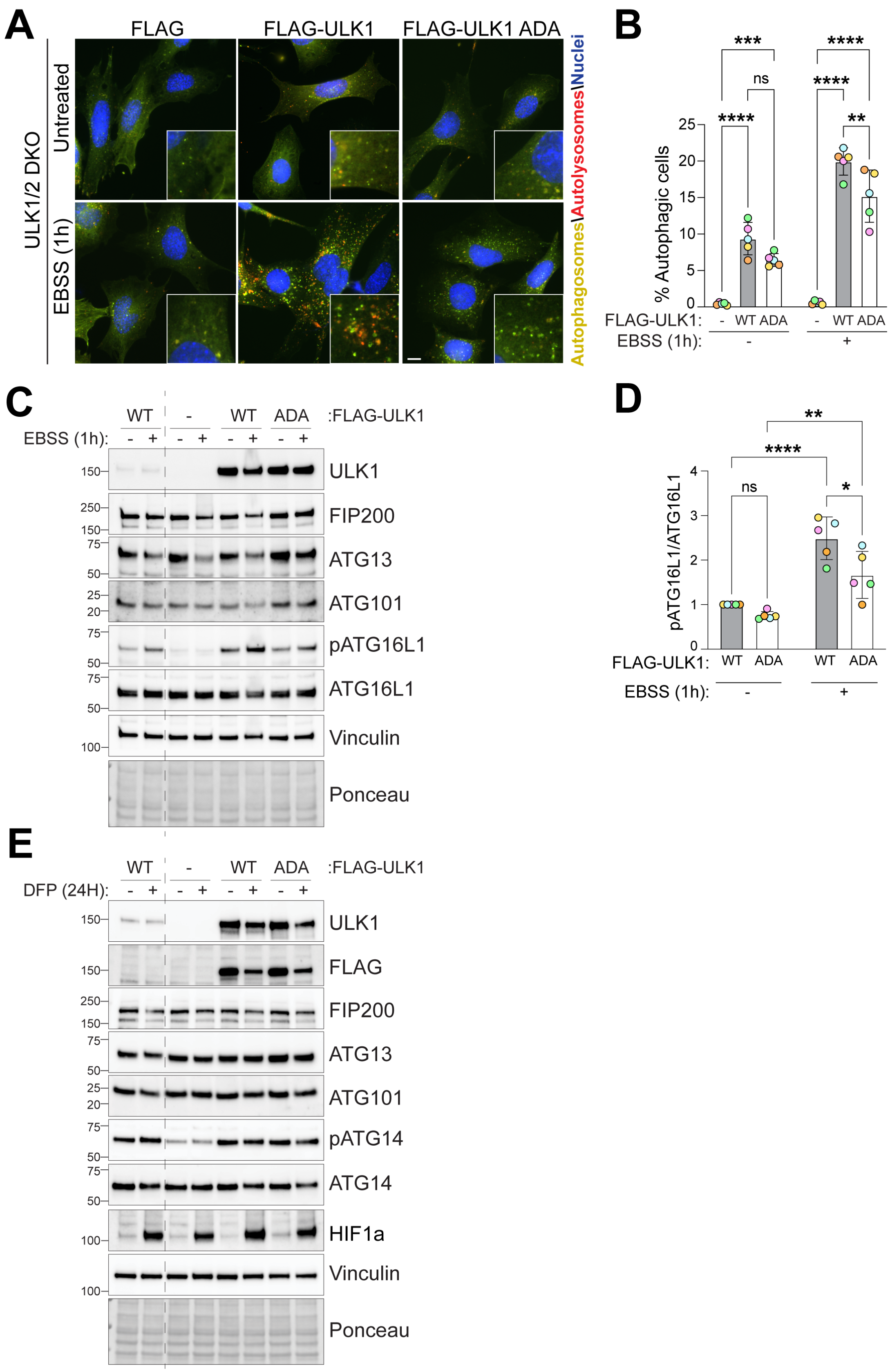

A
FLAG
FLAG-ULK1
FLAG-ULK1 ADA
ULK1/2 DKO
Untreated
EBSS (1h)
Autophagosomes\Autolysosomes\Nuclei
B
% Autophagic cells
FLAG-ULK1: - WT ADA - WT ADA
EBSS (1h): - +
ns
C
WT - WT ADA :FLAG-ULK1
EBSS (1h): - + - + - + - +
ULK1
FIP200
ATG13
ATG101
pATG16L1
ATG16L1
Vinculin
Ponceau
D
pATG16L1/ATG16L1
FLAG-ULK1: WT ADA WT ADA
EBSS (1h): - +
ns
E
WT - WT ADA :FLAG-ULK1
DFP (24H): - + - + - + - +
ULK1
FLAG
FIP200
ATG13
ATG101
pATG14
ATG14
HIF1a
Vinculin
Ponceau

Figure S10: Effect on ULK1 ADA mutation on autophagy and mitophagy

(**A**) Representative wide-field images of ULK1/2 double-knockout (DKO) MEFs stably expressing an empty vector, WT, or ADA mutant FLAG-tagged ULK1 and the Auto-*QC* reporter. Cells were treated with EBSS for 1 hour prior to imaging. **(B)** Flow cytometry analysis of the mCherry/GFP ratio (n = 5 biological replicates); statistics: two-way ANOVA with Tukey's multiple-comparisons test. **(C, D)** Representative immunoblots and quantification of the indicated proteins from cells as in (A) (n = 5 biological replicates); statistics: two-way ANOVA with Tukey's multiple-comparisons test. (**E**) Representative immunoblots of the indicated proteins in lysates of ARPE-19 WT cells or ULK1 knock-out (KO) ARPE-19 cells stably expressing an empty vector (FLAG), WT or ADA mutant FLAG tagged ULK1. Cells were treated with 1mM DFP for 24 hours. Data information: Enlarged images are shown in the lower corners. Nuclei were stained in blue (Hoechst) and scale bars: 10 µm. All data are mean ± s.d.; Statistical significance is displayed as *P ≤ 0.05; **P ≤ 0.01; ***P ≤ 0.001; ****P ≤ 0.0001; ns, not significant.

**Table S1**				
Resourse Type	Resource Name	Source or reference	Identifier	New/Reuse
Dataset	ULK1C structural ensembles	This study	https://zenodo.org/uploads/18778578	New
	ATG13-ATG101-WIPI3 MD trajectory, initial structure, .tpr file	This study	https://zenodo.org/uploads/18778578	New
	ATG13-ATG101-WIPI3-WIPI2 MD trajectory, initial structure, .tpr file	This study	https://zenodo.org/uploads/18778578	New
	Raw data	This study	https://zenodo.org/uploads/18778578	New
Software/Code	ImageLab (ver. 5.2.1)	Bio-Rad Laboratoties, Hercules, CA, USA	RRID:SCR_014210	Reuse
	ChimeraX (ver 1.10)	PMID: 32881101	RRID:SCR_015872	Reuse
	AlphaFold (Ver. 2)	PMID: 34265844	RRID:SCR_023662	Reuse
	Fiji	National Institutes of Health	RRID:SCR_002285	Reuse
	NIS-Elements	Nikon Instruments Inc	RRID:SCR_014329	Reuse
	FACSDiva™	BD, Biosciences	n/a	Reuse
	FlowJo (Ver.11)	BD Biosciences	RRID:SCR_008520	Reuse
	Graphpad Prism (Ver.10)	GraphPad Software, Boston, MA , USA	RRID:SCR_002798	Reuse
	MDAnalysis 2.0.0	https://www.mdanalysis.org/	RRID:SCR_025610	Reuse
	VMD 1.9.4	https://www.ks.uiuc.edu/Research/vmd/	RRID:SCR_024368	Reuse
	ColabFold	https://github.com/sokrypton/ColabFold	RRID:SCR_025453	Reuse
	DeepMSA2	https://aideepmed.com/DeepMSA/	n/a	Reuse
	Adobe Photoshop	https://www.adobe.com/products/photoshop.html	Adobe Photoshop (RRID:SCR_014199)	Reuse
	FACSDiva	https://www.bdbiosciences.com/en-us/products/software/instrument-software/bd-facsdiva-software	BD FACSDiva Software (RRID:SCR_001456)	Reuse
	GROMACS 2021.5	https://manual.gromacs.org/documentation/2021.5/download.html	RRID:SCR_014565	Reuse
	Quantification for beads binding assay	This study	Github: https://doi.org/10.5281/zenodo.20076398	New
Online service	MMseqs2	PMID: 30615063	RRID:SCR_022962	Reuse
	WebLogo	PMID: 15173120	RRID:SCR_010236	Reuse
	CHARMM-GUI	PMID: 18351591	RRID:SCR_025037	Reuse
	HCG web application	https://bio-phys.pages.mpcdf.de/hcg-from-library/	https://github.com/bio-phys/hierarchical-chain-growth/tree/multiprocessing_hcg	Reuse
Plasmid	pCAG-GST-ATG13 (6-196)	PMID: 40442316	RRID:Addgene, 203559	Reuse
	pCAG-GST-TEVs-WIPI3	PMID: 40715440	RRID:Addgene, 223800	Reuse
	pCAG-GST-TEVs-WIPI4	PMID: 40715440	RRID:Addgene, 223801	Reuse
	pCAG-FIP200(1-640)-TSF	PMID: 40442316	RRID:Addgene, 203545	Reuse
	pCAG-ATG101	PMID: 32516362	RRID:Addgene, 189590	Reuse
	pCAG-TSF-ATG101	PMID: 40442316	RRID:Addgene, 210858	Reuse
	pBMN-HA-ATG13	PMID: 37207627	RRID:Addgene, 186223	Reuse
	pLVX mCherry-GFP-mtFIS1(101-152)	MRC PPU Regeants and Services	DU55501	Reuse
	pCMV-VSV-G	Addgene	RRID: Addgene: 8454	Reuse
	GAG/POL	Addgene	RRID: Addgene: 14887	Reuse
	pCA-GFP-ATG13(1-230)	PMID: 33893090	RRID:Addgene, 171413	Reuse
	pCAG-TSF-ULK1-ADA	This study	RRID:Addgene, 249534	New
	pCAG-TSF-ULK1	This study	RRID:Addgene, 249533	New
	pCAG-MBP-ULK1-1-829-mcherry	This study	RRID:Addgene, 249729	New
	pCAG-MBP-ULK1-1-290-mcherrry	This study	RRID:Addgene, 249732	New
	pCAG-MBP-ULK1-290-829-mcherry	This study	RRID:Addgene, 249733	New
	pBMN-HA-ATG13 -HF|DD	This study	RRID:Addgene, 249535	New
	pCAG-TSF-GFP-WIPI3	This study	RRID:Addgene, 257242	New
	pCDH1-HA-ATG101-WT	This study	RRID:Addgene, 257243	New
	pCDH1-HA-ATG101-WF-DD	This study	RRID:Addgene, 257244	New
	pBABE puro mCherry-GFP-FIS 101-152 (mito-QC)	This study	MRC-PPU Reagents and Services, University of Dundee (DU55502)	Reuse
	pBABE puro mCherry GFP LC3	This study	MRC-PPU Reagents and Services, University of Dundee (DU55696)	Reuse
	pBabeD Hygro FLAG	This study	MRC-PPU Reagents and Services, University of Dundee (DU37214)	New
	pBabeD hygro FLAG ULK1	This study	MRC-PPU Reagents and Services, University of Dundee DU45617)	New
	pBabeD hygro FLAG ULK1 P433A V434D P435A	This study	MRC-PPU Reagents and Services, University of Dundee (DU79458)	New
	pBabe puro-FLAG ATG13 siRNA resistant	This study	MRC-PPU Reagents and Services, University of Dundee (DU79579)	New
	pBabe puro-FLAG ATG13 H214D F215D siRNA resistant	This study	MRC-PPU Reagents and Services, University of Dundee (DU79578)	New
Reagent	Glutathione Sepharose 4B GST-tagged protein purification resin	GE healthcare, Chicago, IL	Cat#17075605	Reuse
	Amylose Resin	New England Biolabs, Ipswich, MA	Cat#E8021L	Reuse
	Strep-Tactin Superflow high capacity 50% suspension	IBA Lifesciences, Göttingen, Germany	Cat# 2-1208-010	Reuse
	DMEM	Gibco	Gibco, 10566016	Reuse
	FreeStyle 293 Expression Medium	Thermo Fisher Scientific	12338-026	Reuse

	FBS	Thermo Fisher Scientific	A52568-01	Reuse
	Antibiotic-Antimycotic	Thermo Fisher Scientific	15240062	Reuse
	HEPES	Thermo Fisher Scientific	15630080	Reuse
	PBS	Thermo Fisher Scientific	14190094	Reuse
	DMEM/F-12	Thermo Fisher Scientific	11320033	Reuse
	L-Glutamine	Thermo Fisher Scientific	25030081	Reuse
	Penicillin-Streptomycin	Thermo Fisher Scientific	15140122	Reuse
	Polybrene	Sigma	TR-1003-50UL	Reuse
	Hygromycin B	Thermo Fisher Scientific	10687010	Reuse
	Puromycin	Sigma	P9620-10ML	Reuse
	polyethelenimine	Polysciences	24765	Reuse
	D-Desthiobiotin	Sigma	71610-M	Reuse
	DOPC	Avanti	850375C	Reuse
	DOPS	Avanti	840035C	Reuse
	18:1 PI(3)P	Avanti	850150P	Reuse
	DOPE	Avanti	850725P	Reuse
	Atto647-DOPE	Sigma	42247	Reuse
	SuperSignal™ West Femto Maximum Sensitivity Substrate	Thermo Fisher Scientific	34094	Reuse
	Janelia Fluor® 549 HaloTag® Ligand	promega	GA1110	Reuse
	DFP	Sigma	379409	Reuse
	Ponceau S	Sigma	P3504	Reuse
	EBSS	Gibco	14155	Reuse
	Pierce™ Anti-HA Magnetic Beads	Thermo Fisher Scientific	88836	Reuse
	Lipofectamine 2000	Thermo Fisher Scientific	11668019	Reuse
	Hoechst 33258 solution	Sigma	94403	Reuse
	Bovine Serum Albumin Fraction V	Roche	10735078001	Reuse
	Formaldehyde	Sigma-Aldrich	P6148	Reuse
	cOmplete™, EDTA-free Protease Inhibitor Cocktail	Roche	11873580001	Reuse
	ProLong™ Gold Antifade Mountant	Thermo Fisher Scientific	P36934	Reuse
	Prolong Glass Antifade Mountant	Invitrogen	P36984	Reuse
	MES running buffer	Formedium	MES-SDS5000	Reuse
	BCA assay kit	Thermo Fisher Scientific	23227	Reuse
Cell Line	HEK GnTi	ATCC	(RRID:CVCL_A785)	Reuse
	HeLa S3 cell	ATCC	RRID: CCL-2.2	Reuse
	ARPE-19	ATCC	(ATCC Cat# CRL-2302, RRID:CVCL_0145)	Reuse
	HeLa ATG13 KO cells	This study	RRID:CVCL_F1YY	New
	ARPE-19 ULK1 KO cells	This study	RRID:CVCL_F1YP	New
	HeLa ATG13 KO+HaloTag-LC3 cells	This study	RRID: CVCL_F1YZ	New
	HeLa ATG13-KO+Halo-LC3+HA-ATG13-WT Cells	This study	RRID: CVCL_F1Z0	New
	HeLa ATG13-KO+Halo-LC3+HA-ATG13-HF:DD	This study	RRID: CVCL_F1Z1	New
	HeLa ATG101 KO+HaloTag-LC3 cells	This study		New
	HeLa ATG101-KO+Halo-LC3+HA-ATG101-WT Cells	This study		New
	HeLa ATG101-KO+Halo-LC3+HA-ATG101-WF:DD Cells	This study		New
	Lenti-X™ 293T Cell Line	Takara	Takara,#632180	Reuse
	ULK WT and ULK1/2 double knockout MEFs	From Prof. Craig Thompson, Memorial Sloan-Kettering Cancer Center		Reuse
	293FT	Cellosaurus	RRID:CVCL_6911	Reuse
Protocol	Modeling, MD simulation, and analysis	This study	http://dx.doi.org/10.17504/protocols.io.kqdg31o41l25/v1	New
	Generation of ULK1C structural ensembles	This study	http://dx.doi.org/10.17504/protocols.io.n2bvjemkngk5/v1	New
	Microscopy-based bead protein–protein interaction assay	This study	dx.doi.org/10.17504/protocols.io.4r3l21jojg1y/v1	New
	Membrane reconstitution assay	This study	F0AA720A58A9FEAC02	New
	Halo assay to assess autophagy	This study	F0AA0C0A58A9FEAC02	New
	GUV Reconstitution and Swelling	This study	0AA720A58A9FEAC02	New
	GUV Experiment	This study	F0AA0C0A58A9FEAC02	New
	Purification of proteins	This study	0AA0C0A58A9FEAC02	New
	Mitophagy assay	This study	zp/v1	New
	Cell culture	This study	dx.doi.org/10.17504/protocols.io.bp2l6ex2zgqe/v1	New
	Stable cell lines	This study	F0BBA60A58A9FEAC02	New
	Plasmids construction	This study	0B4C20A58A9FEAC02	New
	Western blot	This study	dx.doi.org/10.17504/protocols.io.x54v9bprzl3e/v1	New
	Immunocytochemistry	This study	/v1	New
	Flow cytometry analysis	This study	F0B4C20A58A9FEAC02	New
	Detection of ATG13-WIPI3 Interaction in Stable Cell Lines	This study	k5/v1	New
	Detection of FLAG ULK1 and Endogenous ATG13 Interaction	This study	3e/v1	New
	SUV production	This study	https://www.protocols.io/private/16B7F0E0C05811F0AA720A58A9FEAC02	New
Antibody	anti-HIF1α	R&D system	R&D system MAB1536, RRID:AB_2116983	Reuse
	anti-HSP60	CST	CST #4870S, RRID:AB_2295614	Reuse
	anti-OMI	MRC PPU Regeants and Services	Cat# S802C	Reuse
	anti-vinculin	Abcam	Abcam ab129002	Reuse
	anti-COXIV	CST	CST #4850S, RRID:AB_2085424	Reuse
	anti-FLAG HRP	Sigma	Sigma, A8592 RRID:AB_439702	Reuse
	anti-FLAG	Sigma	Sigma, F1804 RRID:AB_257946	Reuse
	anti-Phospho-Atg14 (Ser29)	CST	CST #92340 RRID:AB_2800182	Reuse
	anti-Atg14	CST	CST #96752S RRID:AB_2737056	Reuse
	anti-FIP200	Proteintech	Proteintech, 17250-1-AP RID:AB_10666428	Reuse

	anti-p-ATG16L1(S278)	Abcam	Abcam, ab195242	Reuse
	anti-p-ATG16L1(S278)	CST	CST#45511S	Reuse
	anti-ATG16L1	Abcam	Abcam ab187671	Reuse
	anti-ATG13	Sigma	Sigma SAB4200100 RRID:AB_10602787	Reuse
	anti-ULK1	CST	CST #8054S	Reuse
	anti-ATG101	Sigma	Sigma SAB4200175 RRID:AB_10640756	Reuse
	anti-ATG13	CST	CST, 13468 RRID:AB_2797419	Reuse
	anti-GAPDH	Abcam	Abcam, ab8245, RRID:AB_2107448	Reuse
	anti-HA	CST	CST, 3724 RRID:AB_1549585	Reuse
	goat anti-Rabbit IgG (H+L) HRP conjugate	Thermo Fisher Scientific	Thermo Fisher Scientific 31460, Cat# 31460; RRID:AB_228341	Reuse
	goat anti-mouse IgG (H+L) HRP conjugate	Thermo Fisher Scientific	Thermo Fisher Scientific 31430, Cat# 31430; RRID:AB_228307	Reuse
	Donkey anti-sheep IgG (H+L) HRP conjugate	Thermo Fisher Scientific	Thermo Fisher Scientific A16041, RRID:AB_2534715	Reuse
	goat anti rabbit- Alexa Fluor 568	Life Technologies	Life Technologies, A11012, RRID:AB_2534079	Reuse
Other	MycoAlert Detection kit	Lonza	LT07-318	Reuse
	Superdex™ 75 Increase 10/300 GL	Cytica	29148721	Reuse
	Superdex 200 Increase SEC column, 10/300 GL	Cytica	28990944	Reuse
	4-12% Bis-Tris SDS-PAGE	Thermo Fisher Scientific	NP0322BOX	Reuse
	8 well Lab-Tek II Chambered Coverglass	Thermo Fisher Scientific	155409	Reuse
	Protran® Nitrocellulose membrane	Amersham™	15259794	Reuse
	PVDF membrane	Merck	IPVH00010	Reuse
	Hoechst 33342	Thermo Fisher Scientific	H1399	Reuse
	SuperFrost Microscope Slides	VWR	631-0913	Reuse
	Thermo Scientific Menzel X1000 Round Coverslip 13mm #1.5	Thermo Fisher Scientific	11588492	Reuse
	QIAprep Spin Miniprep Kit	Qiagen	27104	Reuse
